# First-Line Targeted Therapy for Hepatocellular Carcinoma: Role of Atezolizumab/Bevacizumab Combination

**DOI:** 10.3390/biomedicines10061304

**Published:** 2022-06-02

**Authors:** Sri Harsha Tella, Anuhya Kommalapati, Amit Mahipal, Zhaohui Jin

**Affiliations:** Department of Medical Oncology, Mayo Clinic, 200 1st Street SW, Rochester, MN 55905, USA; tella.sri@mayo.edu (S.H.T.); kommalapati.anuhya@mayo.edu (A.K.); mahipal.amit@mayo.edu (A.M.)

**Keywords:** liver cancer, immunotherapy, atezolizumab, bevacizumab, nivolumab

## Abstract

Hepatocellular carcinoma (HCC) is an aggressive malignancy accounting for 90% of primary liver malignancies. Therapeutic options for HCC are primarily based on the baseline functional status, the extent of disease at presentation and the underlying liver function that is clinically evaluated by the Barcelona-Clinic Liver Cancer system and Child–Pugh score. In patients with advanced HCC, the United States Food and Drug Administration (US-FDA) approved systemic therapies include the combination of atezolizumab–bevacizumab, sorafenib, and lenvatinib in the first line setting while cabozantinib, regorafenib, ramucirumab (in patients with alfa-fetoprotein [AFP] > 400 ng/mL), pembrolizumab, nivolumab, and nivolumab-ipilimumab combination are reserved for patients who progressed on sorafenib. European Medical Agency (EMA) approved the use of atezolizumab–bevacizumab, sorafenib, and lenvatinib in the first line setting, while cabozantinib, regorafenib, and ramucirumab (in patients with alfa-fetoprotein [AFP] > 400 ng/mL) are approved for use in patients that progressed on first-line therapy. In the first line setting, sorafenib demonstrated a median overall survival (OS) benefit of 3 months as compared to that of best supportive care in randomized phase III trials, while lenvatinib was shown to be non-inferior to sorafenib. Recently, phase 3 studies with immunotherapeutic agents including atezolizumab plus a bevacizumab combination and tremelimumab plus durvalumab combination demonstrated a better OS and progression free survival (PFS) compared to sorafenib in the first-line setting, making them attractive first-line options in advanced HCC. In this review, we outlined the tumorigenesis and immune landscape of HCC in brief and discussed the role and rationale of combining immunotherapy and anti-VEGF therapy. We further expanded on potential limitations and the future directions of immunotherapy in combination with targeted agents in the management of advanced HCC.

## 1. Introduction

Hepatocellular carcinoma (HCC) is an aggressive malignancy accounting for 90% of primary liver malignancies. It is the fourth most common cause of cancer-related deaths in the United States with a median overall survival of 6–20 months, depending upon patient demographics and tumor characteristics [1]. The heterogeneous prognosis in HCC is attributed to the etiology, underlying liver function, and stage of the disease at presentation. While the vaccination of hepatitis B and effective treatment of hepatic viruses helped in decreasing the incidence of HCC in the Eastern World, the incidence of HCC is increasing in the Western World, possibly secondary to metabolic causes [2,3]. Chronic inflammation of hepatic tissue stemming from either genetic or environmental risk factors such as parasitic and viral infections (hepatitis B and C viruses), alcohol, aflatoxins, metabolic syndrome related to obesity, glycogen storage disorders, and diabetes contribute to the tumorigenesis of HCC [4]. Such chronic inflammation promotes immuno-modulatory changes in hepatic tissue, eventually leading to hepatic cirrhosis. This hepatic tissue remodeling explains the presence of cirrhosis in most patients with HCC at the time of initial diagnosis, which is of prognostic importance.

Therapeutic options for HCC are primarily based on the baseline functional status, the extent of disease at presentation and the underlying liver function that is clinically evaluated by the Barcelona-Clinic Liver Cancer system and Child–Pugh score [5]. Surgical resection of the primary tumor remains a mainstay of therapy in patients with localized disease. Yet, surgery is precluded in most patients due to the underlying hepatic cirrhosis or presence of extra-hepatic disease. When surgical resection is not practically feasible, liver-directed therapies such as trans-arterial chemoembolization (TACE), radio-frequency ablation (RFA), stereotactic body radiation therapy (SBRT), and portal vein embolization (PVE) are considered in appropriate clinical contexts [5]. Orthotopic liver transplantation is another possible therapeutic option in patients with unresectable primary tumor. Milan criteria, which consists of an intra-hepatic solitary tumor without macro-vascular invasion and size limits of <5 cm, or three individual tumors each measuring < 3 cm, is generally used as a standard guideline for orthotropic liver transplantation in the United States [6,7]. Using the Milan criteria, several reports have found excellent 5-year survival rates of ≥70% or greater with liver transplantation in the setting of HCC [7,8]. Unfortunately, 15% of patients recur after liver transplant and most patients progress after liver directed therapy. Data from in vitro models demonstrated that the evasion of intrinsic immune system pathways such as the overexpression of vascular endothelial growth factor (VEGF), upregulation of myeloid-derived suppressor and regulatory T (Treg) cells, and suppression of NK-T cells have been implicated in the tumorigenesis of HCC, making anti-VEGF agents and immune check point inhibitors attractive options in HCC [1]. In patients with advanced or extra-hepatic HCC, the United States Food and Drug Administration (US-FDA, Silver Spring, USA)-approved systemic therapies include, atezolizumab–bevacizumab combination, sorafenib, and lenvatinib in the first line setting while cabozantinib, regorafenib, ramucirumab (in patients with alfa-fetoprotein [AFP] > 400 ng/mL), pembrolizumab, nivolumab (the drug manufacturer voluntarily withdrew the approval indication from the US market due to negative study results in CheckMate 459), and, nivolumab-ipilimumab are reserved for patients who progressed during the first-line therapies. European Medical Agency (EMA) approved the use of atezolizumab–bevacizumab, sorafenib, and lenvatinib in the first line setting while cabozantinib, regorafenib, and ramucirumab (in patients with alfa-fetoprotein [AFP] > 400 ng/mL) are approved for use in patients that progressed during first-line therapy. Pembrolizumab and nivolumab monotherapies were not approved by EMA in the management of advanced HCC due to lack of efficacy in overall survival (OS).

In the first line setting, sorafenib demonstrated a median OS benefit of approximately 3 months as compared to that of best supportive care; and lenvatinib was found to be non-inferior to sorafenib in randomized phase III trials [9,10,11,12]. Recently, a phase III trial demonstrated a better OS and progression-free survival (PFS) with atezolizumab–bevacizumab as compared to sorafenib in the first-line setting, making it an attractive option in advanced HCC [13]. Table 1 summarizes the key clinical trials and the medications approved by the US FDA in the management of HCC [9,11,12,13,14,15,16,17,18,19,20,21]. In this review, we outlined the tumorigenesis and immune landscape of HCC in brief and discussed the role and future directions of immunotherapy in combination with targeted therapy in the management of advanced HCC.

## 2. Angiogenesis and Immune Landscape in HCC: The Rationale of Combining Immunotherapy and Anti-VEGF Therapy

HCC often occurs in patients with chronic inflammatory conditions and cirrhosis. The chronic inflammation and fibrotic changes cause low and high-grade dysplasia that eventually cause stromal invasion, leading to HCC [22]. Next generation sequencing studies have shown that, on average, HCC harbors 20–100 mutations/genome. Certainly, the number of mutations per genome differs based on the etiology. For instance, tumors with hepatitis B etiology were known to have higher mutations per genome as the viral DNA incorporates into human genome, leading to chromosomal instability while hepatitis C virus causes chromosomal breaks without integrating to human DNA [23,24]. Mutations in chromatin remodeling genes (*ARID1A/D2*), *TP53, BCL-6, CTNNB1*, *TERT*, genes involved in Janus-kinase (JAK/STAT) and mitogen-activated protein kinase (RAS/MAPK) pathways have been implicated in the tumorigenesis of HCC [25]. In addition, the activation of Wnt/ beta-catenin signaling pathway and phosphorylation of hepatocyte growth factor (HGF), c-Met, and hepatocyte growth factor (HGF) also activate the downstream MAPK pathways, thereby promoting tumorigenesis. As such, HCC tumorigenesis provides a complex interplay of various signaling pathways eventually leading to increased proliferation and decreased apoptosis [25,26,27].

The rapid proliferation of tumor cells leads to local tissue hypoxia stimulating the release of VEGF from the hypoxic or anoxic tumor cells. VEGF not only induces angiogenesis that helps in tumor invasiveness and metastasis, but also helps in recruiting and inducing regulatory T-cells (Tregs), tumor associated macrophages (TAMs), and myeloid-derived suppressor cells (MDSCs). In vitro studies have shown that VEGF has an inhibitory effect on the growth and maturation of dendritic cells, thereby limiting their antigen presenting capacity to CD8+ T-cells. Moreover, VEGF-induced abnormal angiogenesis suppresses the infiltration of CD8+ T-cells into the tumor tissue. Furthermore, TAMs and MDSCs potentiate the immune suppressive environment by releasing immune suppressive cytokines such as IL-10 and tumor growth factor-β, leading to immune escape. VEGF disrupts the typical cancer cell-immunity cycle that comprises cancer cell antigen presentation by dendritic cells to CD8+ T cells, which stimulate further CD8+ T cell infiltration. Hence, VEGF augments the physiological immune suppressive environment caused by hepatic interstitial cells [27]. The combination of immunotherapy and anti-VEGF therapy thus provides an excellent theoretical rational of breaking this VEGF and immune suppressive cycle, thereby promoting the innate immune system to attack the cancer cells. Blocking VEGF by anti-VEGF therapy restores dendritic cell maturation and suppresses abnormal angiogenesis, thereby augmenting the recruitment of CD8+ T cells. Furthermore, blocking VEGF prevents the recruitment of TAMs, MDSCs, and Tregs, which are immune suppressive. As such, anti-VEGF agents enhance the ability of immunotherapy in restoring the anti-cancer immunity. Hence, the combination of immune checkpoint inhibitors and anti-VEGF agents restore and augment the anti-cancer immunity by reprogramming the tumor microenvironment in HCC.

## 3. Atezolizumab and Bevacizumab Combination in HCC

### 3.1. The Phase IB GO30140 Study

The combination of atezolizumab and bevacizumab is by far the most promising immunotherapy combination with targeted therapy. The combination was evaluated in two separate arms in a phase IB GO30140 trial (NCT02715531) [28]. One arm (arm A, *n* = 104) evaluated the safety and efficacy outcomes of the combination regimen while another arm (arm F) randomly assigned patients to atezolizumab alone (*n* = 59) or in combination with bevacizumab (*n* = 60) in treatment-naïve patients with unresectable HCC and good performance status. In arm A, after a median follow up of 12 months, ORR was 36% with a complete response (CR) of 12%. The 1-year disease control rate, PFS, and OS were 75%, 38%, and 63%, respectively. These results are extremely impressive given the fact that a majority of the cohort had advanced HCC with macrovascular invasion (53%), AFP ≥ 400 ng/mL (36%), and 71% had extrahepatic spread. In addition, the combination therapy demonstrated a sustained response, with a median duration of response of ≥ 9 months and ≥12 months in 54% and 30% of the patients, respectively [27]. Hypertension (13%) and proteinuria (7%) were the most common grade 3 or 4 adverse events and 3% (*n* = 3) of the patients suffered treatment-related deaths due to pneumonitis, hepatic dysfunction, and cirrhosis).

Similarly, in arm F, patients with poor prognostic factors were included. For instance, 33% experienced macrovascular invasion and 78% incurred extrahepatic spread and/or macrovascular invasion. After a median follow up of 6.5 months, the combination of atezolizumab (1200 mg) and bevacizumab (15 mg/kg) reduced the risk of death by 45% (Hazard ratio [HR] 0.55 (95% confidence interval [CI]: 0.40–0.74), *p* = 0.01). The PFS was 5.6 months with the combination therapy and 3.4 months in atezolizumab monotherapy. While there were no treatment-related deaths in either groups in arm F, treatment-related serious adverse events were higher (12%) in the combination group as compared to atezolizumab monotherapy (3%). The most common grade 3–4 adverse events noted in the combination therapy were hypertension (5%) and proteinuria (3%). It is important to note that the results of arm F provide the proof-of-concept rationale of combining anti-VEGF therapy with immunotherapy, which makes the tumor microenvironment immune-permissive, thereby improving the efficacy of immunotherapy [27]. With this proof-of-concept rationale and given the ORR of 36%, median PFS and OS of 7 and 12 months (Arm A), respectively, in the phase 1B trial, the combination of atezolizumab and bevacizumab was further evaluated in a phase III trial.

### 3.2. The Phase 3 IMbrave150 Trial

The IMbrave150 (NCT03434379) was a randomized, multicentre, open-label phase III clinical trial that evaluated the co-primary endpoints of OS and PFS benefit (per RECIST 1.1) of atezolizumab 1200 mg-bevacizumab 15 mg/kg as compared to the standard of care, sorafenib in 501 patients with treatment-naïve unresectable HCC [13,29]. The patients were stratified based on geographical location (Asia, except Japan and rest of the World), presence or absence of macrovascular invasion and extra-hepatic spread) and a serum AFP level above or below 400 ng/mL. The trial included a total of 501 patients with advanced HCC, in 69% of initial patients screened for eligibility. Similar high exclusion rates were noticed in other HCC trials. For instance, only 56% and 64% of screened patients were included in CheckMate 459 and REFLECT trials, respectively [9,16]. Among the 501 patients enrolled, 336 were randomized to atezolizumab, among which 38% had macrovascular invasion, 38% had AFP > 400 ng/mL, and 78% had extrahepatic and/or macrovascular invasion indicating that the cohort included patients with advanced disease. Table 2 describes the key inclusion and exclusion criteria of the IMBrave150 trial. Importantly, patients were excluded if they had untreated varices. In case of any adverse event leading to the discontinuation of either agent in the combination therapy, the patients were allowed to continue the other agent if deemed appropriate by the clinical investigator. The combination of atezolizumab and bevacizumab yielded an OS of 85% at 6 months with a corresponding OS of 72% with sorafenib. Similar encouraging results of a better OS with the combination of atezolizumab–bevacizumab were observed at 12 months (67% vs. 55%). It is important to note that these results are from the first interim analysis. Hence, there might be a possibility of overestimation of their OS benefit, especially at the first interim analysis when two interim analyses were planned. On the contrary, the early analysis of OS may prevent the confounding benefit that patients in the sorafenib group may receive from subsequent therapies. It is not uncommon to see a dilution of the OS benefit from posttrial therapies. Nonetheless, the IMbrave 150 trial provided positive results, and the combination therapy was the first to show an OS benefit as compared to sorafenib in advanced HCC. Eventually, an updated analysis at 18 months demonstrated the continued response of the combination therapy (52% with the combination therapy and 40% with sorafenib) [30]. Table 3 outlines the results and response rates in the IMBrave 150 trial [13,29,30].

The sub-group analyses demonstrated a better OS and PFS in the combination arm irrespective of demographic characteristics, performance status, etiological factors, and the extent of advanced disease, including the presence of extra-hepatic disease and macrovascular invasion. Interestingly, the OS and PFS results with the combination therapy were better for patients with viral etiology (hepatitis B or C) as compared to the counterparts without viral etiology. Though not statistically significant, similar favorable results were noted with nivolumab in patients with viral etiology in CheckMate 459 trial [16].

The IMbrave 150 trial evaluated the patient-reported quality of life outcomes using European Organization for Research and Treatment of Cancer (EORTC) quality-of-life questionnaire for cancer (EORTC-QLC-C30). The combination therapy outperformed sorafenib in terms of median time to deterioration of quality of life (11.2 months versus 3.6 months, HR: 0.63 (95% CI: 0.46–0.85, *p* = 0.002). Notably, among other first line agents, namely sorafenib (SHARP trial) and lenvatinib (REFLECT trial), IMbrave150 is the only trial that demonstrated the quality-of-life benefit in patients with advanced HCC [9,12]. Ramucirumab, another VEGF monoclonal antibody that was evaluated in second line setting in advanced HCC, showed non-statistically significant favorable results on quality-of-life analyzed by Functional Assessment of Cancer Therapy Hepatobiliary Symptom Index 8 (FHSI-8) [31].

The combination of atezolizumab and bevacizumab outperformed sorafenib tremendously in terms of safety and the treatment-related grade 3 and 4 adverse events occurred at a higher rate in the sorafenib arm (46%) as compared to the combination of atezolizumab–bevacizumab (36%) (per Common Terminology Criteria for Adverse Events [CTCAE] version 4.0]. The most common adverse events (> 10%) noted in the sorafenib group were diarrhea, palmar-plantar erythrodysesthesia, high blood pressure, hair loss and lack of appetite. Not surprisingly (based on the mechanism of action of drugs), these side effects, except hypertension, were less commonly noted in the combination therapy cohort. Hypertension, proteinuria and elevated liver enzymes were more commonly seen in the atezolizumab–bevacizumab cohort, but the majority of them were of grade 1 or 2 (except hypertension). The dose reduction or interruption rate is marginally higher in the sorafenib group (61%) as compared to the combination of atezolizumab–bevacizumab (49.5%).

While the IMbrave 150 trial opened doors for immunotherapy in HCC, the CheckMate 459 trial left the researchers and clinicians somewhat disappointed with negative results [16]. In CheckMate 459, a total of 743 patients were randomized 1:1 to either nivolumab or sorafenib arm. After a minimum follow of 23 months, nivolumab resulted in a median OS of 16·4 (95% CI: 13·9–18·4) months while median OS with sorafenib was 14·7 (95% CI: 11.9–17.2) months (HR: 0.85; 95% CI 0·72–1·02, *p* = 0.07). Interestingly, OS survival in the sorafenib cohort is the highest so far as compared to prior randomized trials that evaluated sorafenib. Notably, subsequent immune checkpoint inhibitor therapy was administered in 20% of patients who progressed on sorafenib. These results are consistent with single agent durvalumab treatment in a Himalaya trial which demonstrated similar non-significant benefit in OS as compared to that of sorafenib [32]. Additionally, in KEYNOTE-224 trial, pembrolizumab provided durable anti-tumor responses with a median PFS and OS of 4 and 17 months, respectively [17].

### 3.3. Predictive Factors with the Real-World Data

To determine the prognostic factors and real-world outcomes, Korean Cancer Study group performed a retrospective multi-center analysis in patients with unresectable HCC who received the combination of atezolizumab and bevacizumab [33]. Among the 121 patients analyzed, 21% had BCLC stage B disease while the remaining 79% had stage C disease, 37% had macrovascular invasion while 70% had extra-hepatic disease. The median PFS was 6.5 (95% CI: 1.12–4.45, *p* = 0.23) months, OS was not reached, and these results were similar to the data reported in IMbrave150 trial. On performing a multivariate analysis, the authors found that the patients with a neutrophil-lymphocyte ratio (NLR) < 5 yielded a better PFS (HR:2.23, 95% CI: 1.12–4.45, *p* = 0.02) and OS (HR:4.68, 95% CI: 1.87–11.73, *p* < 0.01). Another single-center study from Taiwan also demonstrated the prognostic role of NLR in patients treated with atezolizumab–bevacizumab, but the cut-off used in their study was 3 [34]. A retrospective analysis from Japan evaluated tumor growth kinetics (TGK) and tumor growth rate (TGR) in patients that received atezolizumab and bevacizumab. A total of 88 patients were analyzed and the median PFS of the cohort was 5 months. As per the study protocol, patients with greater than or equal to a 2-fold increase in TGK and TGR were defined as having a hyper-progressive disease. Ten percent of the cohort had a hyper-progressive disease and these patients were found to have a higher intra-hepatic tumor burden, NLR ≥ 3, higher levels of lactate dehydrogenase and AFP [35]. Among all these factors, NLR ≥ 3 was associated with hyper-progressive disease. A few other retrospective single and multi-center analyses have demonstrated the excellent safety and efficacy of atezolizumab–bevacizumab, and the ORR was much higher when the combination regimen was used in first line setting [36,37].

## 4. Future Directions

The single agent immunotherapy trials evaluating pembrolizumab or nivolumab in first-line setting did not yield very encouraging results in terms of OS or PFS advantage as compared to sorafenib, although recently, a couple of phase III trials compared various immunotherapy combinations with sorafenib [32,38]. COSMIC-312 trial evaluated the combination of atezolizumab 1200 mg every 3 weeks and cabozantinib 40 mg/day [38]. The co-primary endpoints were PFS and OS benefit of cabozantinib plus atezolizumab as compared to that of sorafenib. While the study demonstrated the PFS benefit of atezolizumab plus cabozantinib as compared to sorafenib (6.8 months vs. 4.2 months, HR: 0.63; 95% CI: 0.44–0.91, *p* = 0.001), the interim analysis did not demonstrate an OS benefit. The results of the HIMALAYA trial were recently presented during the American Society of Clinical Oncology-Gastrointestinal malignancies (ASCO-GI) 2022 annual symposium [32]. The trial data showed that a combination of dual checkpoint inhibitors, tremelimumab plus a durvalumab combination (STRIDE regimen) showed promising results in terms of a better ORR (5% vs. 20% with sorafenib and STRIDE regimen, respectively) and median OS (13.8 months versus 16.4 months) [32]. However, it is important to note that there was no clinically meaningful difference in OS or PFS in patients receiving single agent durvalumab versus the combination of tremelimumab and durvalumab. As compared to single agent durvalumab, the dual immunotherapy resulted in a better ORR (20% versus 17%) and median duration of response (22 versus 17 months), but marginal benefit was found at the expense of further grade 3/4 treatment-associated adverse events (26% versus 13%). Notably, patients with portal vein thrombosis were excluded, which is a poor prognostic factor. STRIDE regimen, the combination of tremelimumab and durvalumab can thus potentially become the first line treatment option, especially in patients who are at a high risk of bleeding and not eligible to receive bevacizumab. Table 4 summarizes other key clinical trials involving immunotherapy agents in the management of unresectable HCC [16,17,29,38,39,40].

Overall, thus far, the IMbrave150 trial is the only randomized phase III trial that showed a tremendously encouraging response in patients with advanced HCC in all aspects, both primary (OS and PFS) and secondary end points (ORR, safety, and quality-of-life), leading to its approval by medical agencies worldwide. Notably, a few patients were able to achieve complete responses with this combination regimen. The atezolizumab–bevacizumab regimen is considered revolutionary as it was the only first line treatment regimen that demonstrated superior results after the approval of sorafenib more than a decade ago. The encouraging results of the combination therapy directly impacted the future clinical trial design and subsequent use of currently approved therapies in HCC. Many phase III trials are currently exploring the concept of targeting VEGF and immune checkpoint inhibition. A few examples include the combination of pembrolizumab and lenvatinib (NCT03713593), atezolizumab plus cabozantinib (NCT03755791), camrelizumab and apatinib (NCT03764293). It is important to note that all these trials test the efficacy of newer combinations against sorafenib or in patients who progressed or were intolerant to sorafenib. The clinical utility of the combination of immunotherapy and anti-VEGF therapy is yet to be determined in patients who received prior anti-VEGF or immunotherapy. Additionally, the sequence of choosing appropriate second-line agents after progressing to anti-VEGF and immunotherapy combination is challenging as most second-line trials were conducted on patients that progressed on sorafenib. In fact, most approved second-line agents target either VEGF pathway (ramucirumab) or act by checkpoint inhibition (nivolumab, pembrolizumab). Hence, their clinical utility after progressing during anti-VEGF and immunotherapy combination needs to be further evaluated in larger clinical trials. 

It is important to note that about 2/3rd of the patients and more than half of the patients did not respond to single agent immunotherapy (either pembrolizumab or nivolumab) and atezolizumab–bevacizumab therapy, respectively. Interestingly, few patients experienced accelerated progression while on immunotherapy. Unfortunately, no specific biomarker-driven information is available from the IMbrave150 trial. We need a reliable predictive biomarker that could help in maximizing the efficacy of immunotherapy while minimizing the number of patients harmed or not benefitted from immunotherapy combinations. While PD-L1 expression in the tumor cell (TPS) or combined positive scores (assessed as a combination of PD-L1 on tumor and immune cells) are being used as predictive biomarkers in other malignancies such as esophageal, urothelial, and non-small cell lung cancer, PD-L1 CPS ≥ 1% did not correlate with the OS in pembrolizumab (KEYNOTE-224) and nivolumab (CHECKMATE-459) trials [16,41]. It is important to note that the ORR was higher with nivolumab in patients with PD-L1 TPS ≥ 1% as compared to that of PD-L1 < 1% (28% vs. 12%). Despite the better ORR with high PD-L1 expression (≥1%), OS was the same between the two cohorts. This raises the question of specific pattern of progression and response to subsequent therapy based on PD-L1 expression. A subset analysis of KEYNOTE-224 trail showed a correlation of PD-L1 CPS and ORR and PFS [41]. However, the predictive efficacy of CPS is yet to be validated in future randomized trials. Similarly, while there are strong data that support the predictive efficacy of a high tumor mutation burden and high microsatellite instability for pembrolizumab, such a correlation is not encouraging in HCC based on the available data [42]. Pre-clinical data demonstrated that the activation of the Wnt/β-catenin signaling pathway resulted in resistance to immunotherapy [43], but its clinical utility as a predictive biomarker is yet to be determined in larger clinical trials. 

## 5. Conclusions

HCC is the most common primary liver malignancy, and the physiological immune suppression in hepatic tissue and the immune microenvironment plays a key role in its pathogenesis and progression. The data from recent clinical trials involving immunotherapy demonstrated that the dual agent therapy that blocks VEGF and PD-L1 confer survival advantage. As such, the IMbrave150 trial that evaluated the combination of bevacizumab and atezolizumab revolutionized the treatment landscape of HCC with promising results in improving OS with a manageable safety profile. However, at least 50 percent of patients did not respond to the combination regimen, necessitating the requirement of a predictable biomarker. Nonetheless, IMbrave 150 opened the doors for the new era of combination therapies in HCC and many clinical trials evaluating various immunotherapy combinations are underway. We are hopeful that the data from these trials will contribute to the current knowledge and expand our therapeutic strategies in the management of HCC.

## Figures and Tables

**Table 1:** Key phase III clinical trials of US FDA approved therapies in hepatocellular carcinoma.

Study (*n*)	Regimen or Drug(s) Evaluated	Molecular Targets	ORR (%)	mPFS (Months)	mOS (Months)
SHARP (*n* = 602)	Sorafenib 400 mg twice daily compared to placebo	VEGFRs 1–3, PDGFR, RAF, and c-kit	2	4.1	10.7
Asia-Pacific (*n* = 226)			3.3	2.8	6.5
CALGB 80802 (*n* = 356)	Sorafenib 400 mg twice a day (S) compared to sorafenib + doxorubicin (D)		S + D: 9.3 S: 5.4	4 (S + D) 3.9 (S)	9.3 (S + D) 9.4 (S)
REFLECT (*n* = 954)	Lenvatinib 12 mg/day (>60 kg body weight), Lenvatinib 8 mg/day (<60 kg body weight) compared to sorafenib 400 mg twice daily	FGFR 1–4, EGFR 1–3, PDGFR, and c-kit	lenvatinib: 24.1, Sorafenib: 9.2	Lenvatinib: 7.4 Sorafenib: 3.7	Lenvatinib: 13.6 Sorafenib: 12.3
IMbrave 150 (*n* = 501)	Atezolizumab 1200 mg + bevacizumab 15 mg/kg (A + B) compared to sorafenib (S)	PD-L1, VEGF	A + B: 30 S: 11.3	A + B: 6.9 S: 4.3	A + B: 19.2 S: 13.4
CheckMate 459 (*n* = 743)	Nivolumab 240 mg/ 2 weeks (N) * compared to sorafenib 400 mg twice daily (S)	PD-1	N: 15 S: 7	Nivolumab: 3.7 Sorafenib: 3.8	Nivolumab: 16.4 Sorafenib: 14.7
CheckMate 040	Ipilimumab and Nivolumab ^*	PD-1, CTLA4	32% (Arm A) 27% (Arm B) 29% (Arm C)	-	Arm A: 22.8 Arm B: 12.5 Arm C:12.8
KEYNOTE-224 (*n* = 104)	Pembrolizumab *	PD-1	18.3	4.9	13.2
CELESTIAL (*n* = 707)	Cabozantinib 60 mg/day compared to placebo	AXL, MET, and VEGFR2	4	5.2	10.2
RESORCE (*n* = 573)	Regorafenib 160 mg/day compared to placebo	VEGFR 1–3, FGFR, PDGFR, and c-kit	11	3	10.6
REACH (*n* = 565)	Ramucirumab 8 mg/kg compared to placebo	VEGFR-2	7	2.8	9.2
REACH-2 (*n* = 292)			5	2.8	8.5

ORR: Overall Response Rate Per HCC specific modified RECIST criteria; mPFS: median Progression Free Survival of the cohort receiving active agent; mOS: median Overall Survival of the cohort receiving active agent; VEGFR: Vascular endothelial growth factor receptor; RAF: Rapidly accelerated fibrosarcoma; EGFR: Epidermal growth factor receptor; FGFR: Fibroblast growth factor receptor; PDGFR: Platelet-derived growth factor receptor; PD-1/PD-L1: programmed death-ligand 1. ^ Arm A: nivolumab 1 mg/kg plus ipilimumab 3 mg/kg, administered every 3 weeks (4 doses), followed by nivolumab 240 mg every 2 weeks; Arm B: nivolumab 3 mg/kg plus ipilimumab 1 mg/kg, administered every 3 weeks (4 doses), followed by nivolumab 240 mg every 2 weeks; Arm C: nivolumab 3 mg/kg every 2 weeks plus ipilimumab 1 mg/kg every 6 weeks. * Not approved by European Medical Agency for the use in European Union.

**Table 2:** Key inclusion and exclusion criteria of IMbrave 150 trial in patients with hepatocellular carcinoma.

Inclusion Criteria:
- Locally advanced/unresectable or metastatic disease with a minimum of one measurable lesion and no central nervous system metastatic disease or invasion of tumor to major blood vessels or airways - No prior exposure to systemic therapy - Good baseline performance status (ECOG score 0 or 1) - Adequate hematologic and end-organ function without significant medical comorbidities - Child–Pugh class A - No active or bleeding varices on upper GI endoscopy
**Exclusion Criteria:**
- Presence of fibrolamellar or sarcomatoid histologies; co-existing or concurrent malignancies such as cholangiocarcinoma; history of other malignancies with considerable risk of morbidity or death within the last 5 years - Presence or a history of leptomeningeal disease or autoimmune conditions - History or presence of pulmonary disease such as idiopathic pulmonary fibrosis (IPF), drug induced or idiopathic chronic pneumonitis - Infections: Co-existing hepatitis B and C virus infections; Human immunodeficiency virus (HIV) infections - Presence of untreated or partially treated esophageal varices with high risk of bleeding - Pregnancy or breast-feeding females - Worsening cirrhosis leading to moderate to severe ascites and hepatic encephalopathy - Recurrent third spacing of fluids needing frequent drainage procedures - Uncontrolled high blood pressure, bleeding diathesis or coagulopathy, hypercalcemia, or non-healing open wounds - Receipt of liver-directed therapy within the last 28 days or lingering side effects from such procedure performed beyond 28 days - Chronic therapy with non-steroidal anti-inflammatory agents (NSAID)

**Table 3:** Primary and secondary efficacy outcomes in IMbrave 150 trial *.

Results	Atezolizumab and Bevacizumab Combination	Sorafenib	Statistical Significance
**Progression-free survival (months)**	6.9 (95% CI 5.7–8.6)	4.3 (95% CI 4.0–5.6)	HR for disease progression: 0.65; 95% CI: 0.53–0.81; *p* < 0.001
**Overall survival (months)**	19.2 (95% CI: 17.0–23.7)	13.4 months (95% CI 11.4–16.9)	Hazard ratio for death: 0.66; 95% CI: 0.52–0.85; *p* < 0.001
**Overall Response Rate (ORR) per RECIST 1.1 (%)**	29.8 (95% CI: 24.8–35.0)	11.3 (95% CI: 6.9–17.3)	
**CR based on RECIST 1.1 (%)**	8	0.6	
**Disease Control rate (ORR+ stable disease) (RECIST 1.1) (%)**	74	55	
**Median duration of response, months based on RECIST 1.1**	18.1 (95% CI: 14.6-NE)	14.9 (95% CI: 4.9–17.0)	

* After a median 15.6 (range, 0–28.6) months of follow-up.

**Table 4:** Key clinical trials involving immunotherapy agents in first line setting in hepatocellular carcinoma.

Study (*n*)	Regimen or Drug(s) Evaluated	Molecular Targets	ORR (%)	mPFS (Months)	mOS (Months)
IMbrave 150 (*n* = 501)	Atezolizumab 1200 mg + bevacizumab 15 mg/kg (A + B) compared to sorafenib (S)	PD-L1, VEGF	A + B: 30 S: 11.3	A + B: 6.9 S: 4.3	A + B: 19.2 S: 13.4
CheckMate 459 (*n* = 743)	Nivolumab 240 mg/ 2 weeks (N) compared to sorafenib 400 mg twice daily (S)	PD-1	N: 15 S: 7	Nivolumab: 3.7 Sorafenib: 3.8	Nivolumab: 16.4 Sorafenib: 14.7
KEYNOTE-224 (*n* = 104)	Pembrolizumab	PD-1	16	4	17
KEYNOTE-524 (*n* = 104)	Pembrolizumab and lenvatinib	PD-1, multiple kinases (VEGF, FGF, PDGFRα, RET, KIT)	46	9.3	22
COSMIC-312 (*n* = 837)	Atezolizumab 1200 mg + cabozantinib 40 mg or cabozantinib 60 mg compared to sorafenib	PD-L1, multiple kinases (c-Met VEGFR2, AXL, and RET)	−	A + C: 6.8 S: 4.2	−
RESCUE (*n* = 70, first-line setting)	Camrelizumab 3 mg/kg (max: 200 mg) every 2 weeks + apatinib 250 mg	humanized, IgG4-κ PD-1 mAb, VEGFR-2	34.3	5.7	-

ORR: Overall Response Rate Per HCC specific modified RECIST criteria; mPFS: median Progression Free Survival of the cohort receiving active agent; mOS: median Overall Survival of the cohort receiving active agent; VEGFR: Vascular endothelial growth factor receptor; RAF: Rapidly accelerated fibrosarcoma; EGFR: Epidermal growth factor receptor; FGFR: Fibroblast growth factor receptor; PDGFR: Platelet derived growth factor receptor; PD-1/PD-L1: programmed death-ligand 1.
